# Yet Another Flawed “Placebo Controlled” Study in Crohn's Disease?

**DOI:** 10.1089/fpd.2015.1999

**Published:** 2015-09-01

**Authors:** Robert J. Greenstein, D. William Cameron, Sheldon T. Brown

**Affiliations:** ^1^ Department of Surgery, James J. Peters Veterans Affairs Medical Center, Bronx, New York.; ^2^ Department of Medicine, Ottawa Hospital Research Institute, Ottawa, Ontario, Canada.; ^3^ Division of Infections Diseases, James J. Peters Veterans Affairs Medical Center, Bronx, New York.; ^4^ Inchan School of Medicine at Mount Sinai, New York, New York.

Dear Editor:

Accumulating data suggest that some variants of Crohn's disease (CD) may be consequent to a *Mycobacterium avium* subspecies *paratuberculosis* (MAP) infection. Opportunely, a prospective study (Kalfus, 2013) is addressing the therapeutic role of appropriate antimycobacterial antimicrobials in CD. Potential inflammatory bowel disease (IBD) benefits may include identification of a mycobacterial etiology, improvement on current therapies, and possibly prevention.

MAP is inhibited by agents that the scientific community categorizes as anti-inflammatory (Greenstein *et al.,* 2007) and immune-modulators. (Krishnan *et al.,* 2009) A much-cited 2007 “placebo controlled” trial of anti-MAP antibiotics concluded that there was “no significant role for MAP in the pathogenesis of CD” (Selby *et al.,* 2007). Subsequent correspondence dismissed the possible corrupting influence of unanticipated “immune-modulator” anti-MAP inhibition as being “not consistent” with prevailing dogma. This was despite the editorial accompanying the 2007 study emphasizing significant improvement associated with concomitant use of “immune-modulators” (Selby *et al.,* 2007).

Disconcertingly, the inclusion criteria in the ongoing study (Kalfus, 2013) are essentially identical to the prior “placebo”-controlled study (Selby *et al.,* 2007). Both ignore MAP inhibition by “anti-inflammatory” (Greenstein *et al.,* 2007) and “immune-modulators.” (Krishnan *et al.,* 2009) The 2007 study (Selby *et al.,* 2007) was designed before relevant MAP inhibition data (Greenstein *et al.,* 2007; Krishnan *et al.,* 2009) were published. The designers of the study now recruiting (Kalfus, 2013) should explain why they ignore unrefuted published data that render their “placebo-controlled” study as flawed as previously (Selby *et al.,* 2007).

We suggest that calling the study now recruiting (Kalfus, 2013) “placebo-controlled” is unscientific (Greenstein *et al.,* 2014). Multiple medications that inhibit infectious agents (Greenstein *et al.,* 2014) are permitted in both the “placebo” as well as treated groups. (Greenstein *et al.,* 2014). Previously, we have suggested that “add-on studies” is scientifically more accurate than “placebo-controlled” (Greenstein *et al.,* 2014).

A consistently flawed study design may yet again be misinterpreted as showing no added efficacy of anti-MAP agents in CD. Consequently, the potential identification of the etiology of IBD as being due to the zoonosis of MAP may irrevocably be lost.
